# Perfluoroalkyl Mixture Exposure in Relation to Fetal Growth: Potential Roles of Maternal Characteristics and Associations with Birth Outcomes

**DOI:** 10.3390/toxics10110650

**Published:** 2022-10-28

**Authors:** Chensi Shen, Jiaxin Ding, Chenye Xu, Long Zhang, Shuren Liu, Yonghong Tian

**Affiliations:** 1 College of Environmental Science and Engineering, Donghua University, Shanghai 201620, China; 2 Shanghai Institute of Pollution Control and Ecological Security, Shanghai 200092, China; 3 Women’s Hospital, Zhejiang University School of Medicine, Hangzhou 310000, China; 4 Interdisciplinary Research Academy (IRA), Zhejiang Shuren University, Hangzhou 310015, China

**Keywords:** perfluoroalkyl substances, prenatal exposure, maternal determinants, birth weight, apgar scores, preterm birth, mixture effects

## Abstract

Perfluoroalkyl substances (PFASs) exposure is suggested to interfere with fetal growth. However, limited investigations considered the roles of parity and delivery on PFASs distributions and the joint effects of PFASs mixture on birth outcomes. In this study, 506 birth cohorts were investigated in Hangzhou, China with 14 PFASs measured in maternal serum. Mothers with higher maternal ages who underwent cesarean section were associated with elevated PFASs burden, while parity showed a significant but diverse influence. A logarithmic unit increment in perfluorooctanoic acid (PFOA), perfluorooctane sulfonate (PFOS), and perfluorononane sulfonate (PFNS) was significantly associated with a reduced birth weight of 0.153 kg (95% confidence interval (CI): −0.274, −0.031, *p* = 0.014), 0.217 kg (95% CI: −0.385, −0.049, *p* = 0.012), and 0.137 kg (95% CI: −0.270, −0.003, *p* = 0.044), respectively. Higher perfluoroheptanoic acid (PFHpA) and perfluoroheptane sulphonate (PFHpS) were associated with increased Apgar-1 scores. PFOA (Odds ratio (OR): 2.17, 95% CI: 1.27, 3.71, *p* = 0.004) and PFNS (OR:1.59, 95% CI: 1.01, 2.50, *p* = 0.043) were also risk factors to preterm birth. In addition, the quantile-based g-computation showed that PFASs mixture exposure was significantly associated with Apgar-1 (OR: 0.324, 95%CI: 0.068, 0.579, *p* = 0.013) and preterm birth (OR: 0.356, 95% CI: 0.149, 0.845, *p* = 0.019). In conclusion, PFASs were widely distributed in the maternal serum, which was influenced by maternal characteristics and significantly associated with several birth outcomes. Further investigation should focus on the placenta transfer and toxicities of PFASs.

## 1. Introduction

Perfluoroalkyl substances (PFASs) comprise a group of synthetic fluorinated chemicals that have been widely used in cleaning products, textiles, adhesive food packaging, and fire foam for decades [1,2,3,4]. Through water drinking, food consumption, and air inhalation, PFASs chemicals are able to enter the human body, binding to serum albumin and then distributed in the lungs, liver, and brain [5,6]. This is particularly problematic for pregnant women and their newborns since they are vulnerable to exogenous exposure and PFASs are “known to be toxic” [7]. Currently, increasing numbers of studies have detected PFASs in the umbilical cord, maternal serum, placenta, meconium, and fecal samples [8,9,10], which exerted health concerns for the long term.

Among the above matrix, PFASs in maternal serum are commonly used to reveal the prenatal and in-uterus exposure because of the data feasibility and sample availability [11]. For expectant mothers, maternal age, body mass index (BMI), water drinking source, and die features have been widely identified as predictors of PFASs exposure in maternal serum [12,13,14,15,16,17,18,19,20,21,22]. Prior studies also suggested that longer breastfeeding duration might reduce PFASs burden in mothers [16]. A U.S. birth cohort further confirmed that women who were parous, with a previous breastfeeding history, black race, or in alower income bracket had significantly declined geometric mean concentrations of perfluorooctane sulfonate (PFOS) and perfluorooctanoic acid (PFOA) [23]. Among all these possible determinants, whether parity and delivery mode are associated with PFASs accumulation has not been well documented so far.

For fetuses, chemical exposure during gestation periods could adversely affect child and adult growth [14,24,25,26]. In-depth evidence related the fetal growth impairment of reduced birth weight to multiple PFASs exposure [27,28,29,30]. A recent meta-analysis considering 46 epidemiological studies suggested that birth weight was inversely associated with PFASs exposure, with effect sizes ranging from −181.2 g per ng/mL increase in perfluoroheptanesulfonate (PFHpS) to −24.3 g per ln (ng/mL) increase in perfluorodecaoic acid (PFDA) [31]. Preterm birth was defined as <37 completed weeks’ gestation. Gestational exposure to several PFASs compounds was associated with increased odds of preterm birth [31,32,33], however, some prospective cohorts provided null association [34]. For fetal growth considerations, reliance on birth weight and gestation age as the birth anthropometric measurements precludes the determination of physical development associated with PFASs exposure. The activity, pulse, grimace, appearance, and respiration (Apgar score) has emerged as a standard evaluation method for assessing the physical condition of a child, which is conducted 1 and 5 min after birth [35]. Currently, only two studies have examined the effect of PFASs on infant Apgar scores but they showed inconsistent directions [35,36]. Moreover, it is noteworthy that most previous studies in this field have assessed the associations based on individual PFAS chemical exposure, which seems “insufficient” for risk evaluations [32,37,38]. Considering the complex exposure patterns and highly correlated compounds, the evidence regarding the joint effects of PFASs on birth outcomes appears to be a sheer necessity in biomonitoring work.

Given the aforementioned data, this study monitored 14 PFASs in maternal serum from 506 mother-infant pairs in Hangzhou, China. We aimed to (1) measure PFASs profiles in maternal serum before delivery, (2) explore the potential sociodemographic predictors of PFASs exposure, and (3) provide epidemiologic evidence on both joint and individual associations between PFASs exposure and birth outcomes (continuous outcomes of birth weight, Apgar 1 and Apgar 5 as well as the binary outcome of preterm birth).

## 2. Materials and Methods

### 2.1. Study Population, Birth Outcome Ascertainment and Sample Collection

Maternal-neonatal pairs were recruited, and prenatal maternal serum was collected at the Women’s Hospital School of Medicine, Zhejiang University in Hangzhou, China from October 2020 to September 2021. The eligible mothers were older than 20 y and excluded from reporting serious medical treatment history, including neoplastic diseases, cardiovascular diseases, renal failure, aortic surgery, chronic liver failure, gestational hypertension, and other medical conditions [21]. The infants who met the research conditions were singletons and had no congenital diseases. Consequently, the 506 birth cohort was finally included in the survey. Demographic characteristics, including maternal age, prenatal BMI, education, occupation, smoking, alcohol drinking, ethnicity, and parity were collected from structured questionnaires and follow-up medical records. The gestational age was determined based on the last menstrual period [39] and the information on gestational age and mode of delivery was recorded at delivery (Table S1).

Birth weight (kg) and Apgar scores (Apgar-1 and Apgar-5) were collected from the delivery records. The World Health Organization defines preterm birth as birth occurring before 37 completed weeks of gestation [40].

Blood samples were collected at the hospital (with the permission of the Medical Ethics Committee of Women’s Hospital, School of Medicine, Zhejiang University (IRB-20200055-R), and written consent was obtained from each donor) and then centrifuged at 4000 rpm for 20 min to separate and extract the serum. Finally, the serum was transferred to a polypropylene tube and stored at −4 °C for further analysis.

### 2.2. Sample Extraction and Instrument Analysis

Native standards of perfluoroalkyl sulfonic acids (PFSAs), perfluoroalkyl carboxylic acids (PFCAs), and corresponding isotopically labeled internal standards were supplied by Wellington Laboratories (Guelph, ON, Canada). The ammonium hydroxide, high-performance liquid chromatography-grade (HPLC-grade) methanol, and formic acid were purchased from J&K Chemicals (Shanghai, China). A total of 14 PFASs and corresponding internal standards are available in Table S2.

Solid-phase extraction was conducted to extract the PFASs from the serum samples. The procedures are presented in detail in SI. All serum samples were spiked with isotopically labeled internal standards. Cartridges were activated after the precondition. Prepared samples were subsequently passed through the cartridges, which were then washed [41] (Woudneh et al., 2019). Ammonium hydroxide in methanol and methanol were used to elute chemicals. The eluates were collected and evaporated until they were dry and then rediluted in methanol [41,42]. The aforementioned extracts were vortex-mixed and then transferred to polypropylene vials prior to analysis [10].

Compounds were separated using an Acquity UPLC BEH C18 column (2.1 mm × 50 mm, 1.7 μm, Waters, Dublin, Ireland). All 14 target compounds were analyzed on a UPLC-tandem electrospray equipped with the Xevo TQ-Striple quadrupole mass spectrometry system (Waters ACQUITY UPLC I-Class, Milford, MA, USA). The MS parameters of the target analytes are listed in Table S3.

### 2.3. Quality Assurance and Quality Control

All laboratory utensils were polypropylene and moistened with Milli-Q water and HPLC-grade methanol three times before use. To check for potential background contamination between batches, procedure blanks for every 20 samples were included. The PFASs in the present study were quantified using the internal-standard method. Calibration consisted of native PFASs containing internal calibration. The process blank sample, matrix spiked sample, and three groups of parallel samples were repeated twice for each group for the quality control measures in the pretreatment process. The mean matrix spiked recoveries ranged from 86.7% to 107%. The corresponding internal standard, correlation coefficient, detection limit, quantification limit, and method detection limit of each target compound are shown in Table S2. The limits of detection and quantification are defined as the concentrations in the diluted standard solution in response to the signal-to-noise ratio of 3 and 10.

### 2.4. Statistical Analysis

Descriptive statistical analyses, including the mean ± standard deviation (SD) and *n* (%), were selected to express the demographic characteristics. All data were assessed for normality and homogeneity via the Kolmogorov–Smirnov test and the Bartlett test [39]. PFASs concentrations were expressed as medians with interquartile ranges (IQR). The PFASs concentrations showed skewed distributions; thus, the PFASs exposure was log_10_-transformed before further analysis [43]. The Spearman correlation coefficient estimated the correlations between individual PFASs compounds. Nonparametric Mann–Whitney U testing and Kruskal–Wallis analysis were conducted to compare the concentrations of the possible sociodemographic predictors employed to explore the effects of maternal factors on PFASs distributions.

Univariate and multivariate linear regression models were utilized to determine the associations between the concentration of each PFAS compound and birth outcomes. For continuous outcome variables of birth weight and Apgar scores, the results were expressed as estimated changes in each unit per log-unit increase in PFASs in the maternal serum (β and 95% confidence intervals (CI)). For the categorical dependent variable of preterm birth, binary logistic regression models were used to estimate the odds ratios (ORs) and 95% CI for preterm birth and PFASs exposures [32]. For the mixture analysis, a quantile-based g-computation was performed to estimate the joint effects of PFASs in relation to birth outcomes. This novel method combined weighted quantile sum regression and g-computation [44,45], which produces estimates of the simultaneous effect on the overall effects of an increase of exposure mixture by one quantile [46]. In this study, the quantile was set to one quartile increase in log-PFAS concentrations, and each exposure is given its weight and direction [19,46]. Potential covariates were chosen a priori [10,30,31,37,47,48] and a directed acyclic graph (DAG) (Figure S1): maternal age (years), prenatal BMI, education (below high school, college, postgraduates), occupation (employee, self-employment, unemployment), smoking (yes or no), alcohol drinking (yes or no), ethnicity (Han or others), delivery mode (spontaneous labor or cesarean birth) and parity (1, 2, ≥3). *p* < 0.05 was regarded as statistically significant. All analyses were performed using SPSS (Version 22.0; SPSS Inc., Chicago, IL, USA) and R (version 4.2.0) with the “qgcomp” package.

## 3. Results and Discussions

### 3.1. Demographic Characteristics of the Studied Population

The pregnant women included in the study were aged 31.3 (SD = 4.28) y on average at delivery. Prenatal BMI was 26.7 ± 3.16 kg/m^2^, with an average gestation age of 265 ± 28.3 d. About 41.3% were primiparous, and nearly 51.2% of mothers had spontaneous labor. Of the newborns, 52.9% were male. There were 89 neonates born with preterm birth and the percentage was 17.6%. The average birth weight was 3.11 (SD = 0.75) kg and the average neonatal Apgar-1 and Apgar-5 scores were 9.88 and 9.99, respectively. Approximately 2.6% and 2.0% of newborns had Apgar-1 and Apgar-5 scores below 8. All the demographic information is shown in Table S1.

### 3.2. PFASs Distributions in Maternal Serum

**Figure 1:**
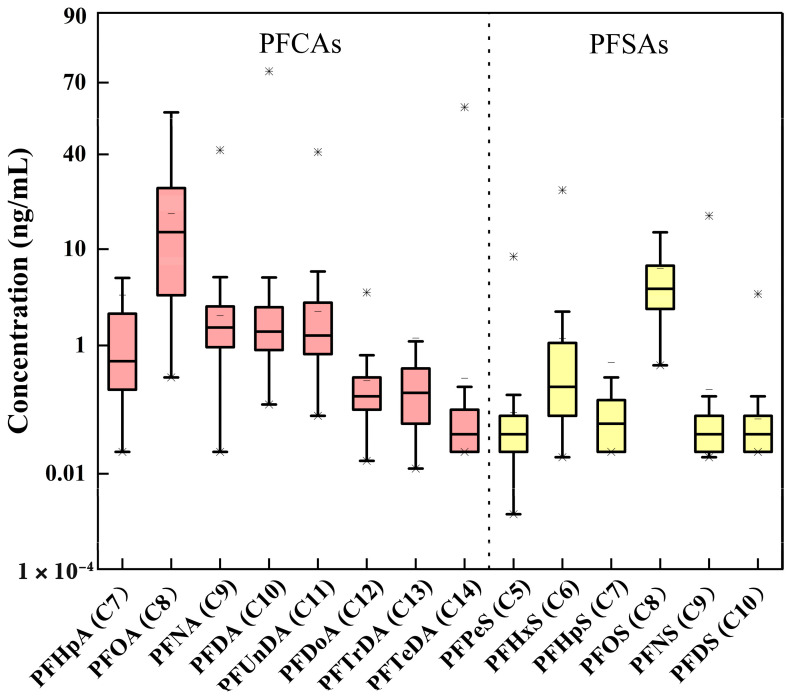
Levels of PFAS (ng/mL) in maternal serum, * means the maximum concentration.

**Figure 2:**
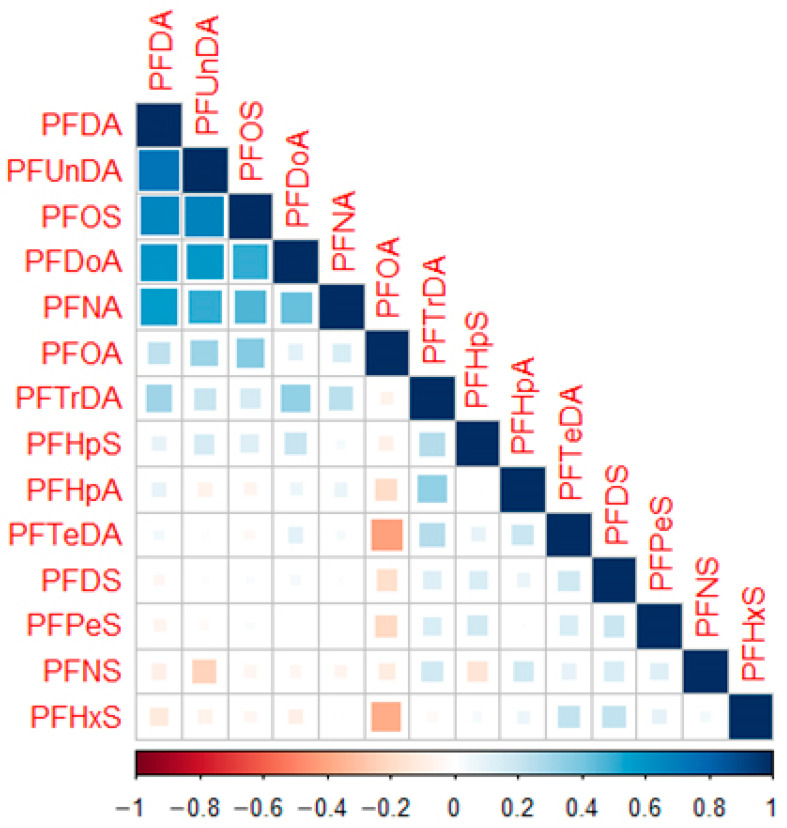
Spearman correlation coefficients for PFASs in serum.

Fourteen PFASs homologs were analyzed in the maternal serum. Except for PFTeDA, PFHxS, and PFHpS, all compounds exhibited high detection frequencies exceeding 80%. As shown in Figure 1, PFOA was the most abundant PFASs, with the median concentration of 13.6 ng/mL, followed by PFOS (4.32 ng/mL) > PFNA (1.66 ng/mL) > PFDA (1.48 ng/mL) > PFUnDA (1.33 ng/mL) > PFHpA (0.625 ng/mL) > PFHxS (0.250 ng/mL) > PFTrDA (0.225 ng/mL) > PFDoA (0.200 ng/mL)> PFTeDA (0.050 ng/mL) ≈ PFDS, PFPeS, PFNS (Table S4). As displayed in Table S5 and Figure 2, significant positive correlations were found among several long-chain PFASs (C ≥ 8 i.e., PFDA, PFUnDA, PFOS, PFDoA, PFNA, PFOA).

Compared with the previous studies in the past three years, the median PFOA concentrations were higher than those of Shenyang, China (3.27 ng/mL) [49], Odense, Denmark (1.7 ng/mL) [50], and Hokkaido, Japan (2.0 ng/mL) [51]. The median PFOS concentrations in this study were comparable to those reported in New Jersey, United States (median, 4.25 ng/mL) [2], slightly higher than that in Hokkaido, Japan (3.4 ng/mL) [51] but lower than that in Hebei, China (7.3 ng/mL) [52] (Wang et al., 2018), Guangdong, China (7.15 ng/mL) [27], Odense, Denmark (7.5 ng/mL) [50], and the United Kingdom (13.8 ng/mL) [53]. For other homologs, PFNA (C9) had the third highest level, comprising 7.58% of the total PFASs. The median concentration of PFNA (1.66 ng/mL) was considerably higher than that reported in Guangzhou, China (0.2 ng/mL) [39] and Beijing, China (0.57 ng/mL) [54]. Different from PFOS and PFOA, PFNA had an elimination rate of 1–2 months and was preferentially stored in the liver [55]. As the major long-chain PFASs (C9–C13), PFNA could have higher acute toxicity and bioaccumulation potential than short-chain PFASs [56].

### 3.3. Potential Roles of Maternal Determinants

Higher maternal age (>35 years) was significantly associated with higher concentrations of PFOA, PFNA, PFDA, PFUnDA, PFOS, and PFPeS than those in the lower age groups (*p* < 0.05) (Table 1). Our finding may be explained by older women having higher cumulative PFAS exposure than other women; thus, older women may have had more PFASs exposure than younger women. This difference was attributable to the biopersistence and long elimination median half-lives of PFASs [21,57,58]. The concentrations of PFHpA, PFOA, and PFHxS in the highestbody mass index (BMI) group were elevated compared to those in the other two groups, whereas women with pre-pregnancy BMI > 25 kg/m^2^ had lower PFNA, PFUnDA, and PFHpS levels than did those with normal BMI (<25 kg/m^2^). In this study, no significant associations between BMI and serum PFAS were noted. Lipophilic persistent organic pollutants can usually accumulate and become enriched in adipose tissue, which was described as the BMI of the mothers. However, mixed results were found for PFASs mainly bound to albumin-positive, null, and inverse associations between BMI and PFASs concentrations [21,59].

Parity played a significant role in PFASs exposure. Primiparous mothers tend to have significantly higher levels of PFDoA and PFTrDA than multiparous ones (*p* < 0.05). Nevertheless, PFOA, PFDA, PFUnDA, PFNA, PFOS, PFNS, and PFHxS exhibited a reverse trend in that the concentrations were significantly higher in multiparous women than in primiparas. It can be seen that parity was associated with higher concentrations of seven PFASs but the differences between nulliparous and parous women became smaller for PFOS, PFNS, PFOA, and PFHpS. In this study, we cannot draw the conclusion that parity was a strong negative or positive predictor of maternal PFAS status for individual differences between study subjects. However, a prior study has reported that a Swedish birth cohort observed that parity was inversely associated with eight serum PFASs except for PFOS, PFNA, and PFOA concentrations declined as parity increased [60]. Breastfeeding and delivery were identified as the main pathways for PFASs excretion and the elimination by lactation and placental transfer related to lowering PFASs exposure [58,61,62]. However, some other studies suggested that the effects of lactation and childbirth have gradually disappeared, and the PFASs levels have returned to the prenatal concentrations [63].

In addition, the current study firstly explored the association between delivery modes and PFASs exposure. The concentrations of PFOA, PFDA, PFUnDA, PFOS, PFHxS, and PFPeS were significantly higher in mothers who underwent cesarean section (C-section) than in those who had spontaneous delivery. In this study, the majority of pregnant women who delivered their babies via C-section were in the elderly group, who tend to accumulate PFASs for a longer time. The poor contractile force and guiding extension tension of the uterus of elderly pregnant women can easily prolong the delivery time, and the fetus delivered via C-section also had the problem of excessive birth size.

### 3.4. Associations between PFASs Exposure and Birth Weight and Apgar Scores

**Figure 3:**
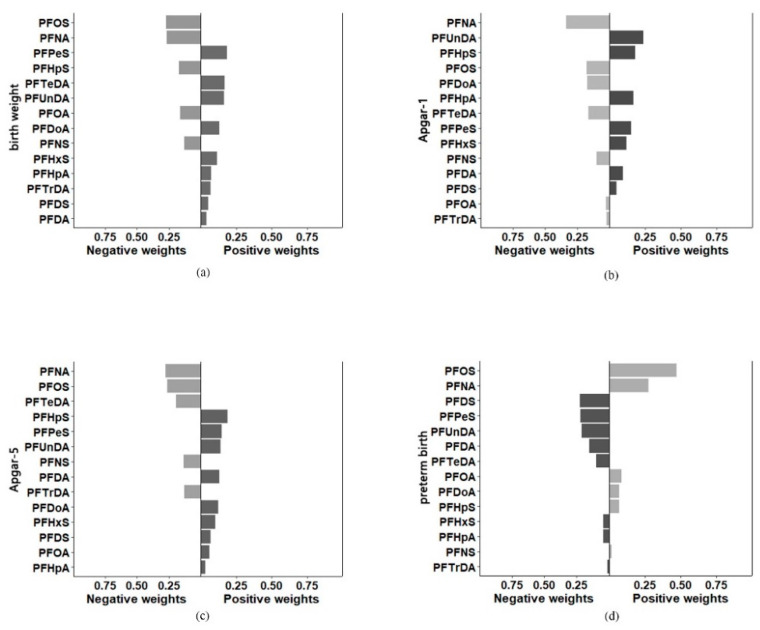
Quantile-based g-computation approach of PFAS on birth outcomes. (**a**) birth weight, (**b**) Apgar-1, (**c**) Apgar-5, (**d**) preterm birth. Note: PFAS were log-transformed and missing data imputed. The model was adjusted for maternal age, prenatal BMI, education, occupation, smoking, alcohol drinking, ethnicity, delivery mode and parity.

Univariate linear regression results suggested that serum PFOA, PFOS, and PFNS were inversely associated with birth weight (Table 2). Specifically, each logarithmic unit increment in PFOA, PFOS, and PFNS was significantly related to the following reduced birth weights, respectively: 0.153 kg (95% CI: −0.274, −0.031, *p* = 0.014), 0.217 kg (95% CI: −0.385, −0.049 *p* =0.012), and 0.137 kg (95% CI: −0.270, −0.003 *p* = 0.044). When adjusted for all covariates, the negative associations remained but were not significant for PFOA: −0.110 kg (95%CI: −0.232, 0.012). Moreover, PFNA, PFUnDA, PFTeDA, and PFHpS exhibited a negative correlation with birth weight in both models, although these data were not significant. Given that PFASs had strong inter-correlations, we, therefore, performed quantile-based g-computations to further explore the joint effects of PFASs on birth outcomes. However, there was no significant association between the PFASs mixture and birth weight (Figure S2a and Table 3). By weight in decreasing order, PFOS, PFNA, PFHpS, PFOA, and PFNS had a negative influence on the total PFASs mixture estimate on birth weight, while other PFASs exhibited positive effects (Figure 3). Birth weight can be adversely affected by prenatal PFASs exposure in utero [64]. Previous epidemiological evidence suggested higher PFASs (particularly PFOA and PFOS) exposure in relation to lower birth weights in newborns [6,28,47,65]. Several mechanisms were proposed to explain the negative correlation. Firstly, fetal reproductive hormones, such as estradiol, total testosterone, and progesterone, could be affected by in utero exposure to PFOS and PFOA, these substances could adversely affect fetal development [66]. Furthermore, PFASs exposure obstructed trophoblast cell proliferation, indicating a possible association between prenatal PFASs exposure and adverse placentation [67].

With regard to Apgar scores (activity, pulse, grimace, appearance, and respiration), in the fully adjusted models, per logarithmic unit increase in PFHpA and PFHpS exposure was associated with increased Apgar-1 scores of 0.065 (95% CI: 0.002, 0.129, *p* = 0.044) and 0.117 (95% CI: 0.006, 0.228, *p* = 0.039). Quantile g-computation model indicated that increasing all PFASs in the mixture by one quartile was significant with a 0.324 increase in Apgar-1 (95% CI: 0.068, 0.579, *p* = 0.013) (Figure S2b). However, no significant association was found between PFASs and Apgar-5, neither individual nor joint exposure (Figure S2c). To date, only two studies have thus far investigated the associations between PFASs exposure and Apgar scores. A birth cohort reported a decrease in the mean Apgar score in logarithm-transformed PFOA concentrations (β: −1.37, 95% CI: −2.42 to −0.32) [35]. Another birth cohort study from Denmark indicated that the odds ratios for Apgar score <10 were 1.20 (95% CI: 0.67, 2.14) and 1.14 (95% CI: 0.57, 2.25) for higher PFOS and PFOA exposures [36]. These two studies draw the conclusion that PFOS and PFOA could enhance the probability of score reduction, but it is not the case in this study. It was noteworthy, as two shorter chain PFASs than C8-PFASs, PFHpA and PFHpS were thought to be less bioaccumulative and have comparably lower toxicity profiles. Positive associations were previously found for PFHpA, PFOA, PFHpS, and PFOS and lower respiratory tract infections (LRTI) [68]. Findings from the Shenyang birth cohort indicated that PFHpA was the important contributor (45.0%) among the PFASs mixture to the decrease of thyroid stimulating hormone (TSH) levels of newborns [49]. A negative association between PFHpA and luteinizing hormone (LH) and free androgen index (FAI) was also previously confirmed [69]. Since there is no previous information discussing the effects of PFHpA and PFHpS on the Apgar scores, and they were not a major contributor to total PFASs concentrations in maternal serum, the reproductive toxicity of whether these two short-chain PFASs exerted the health effects on the fetal growth remains uncertain.

### 3.5. PFASs Exposure in Relation to Preterm Birth

**Figure 4:**
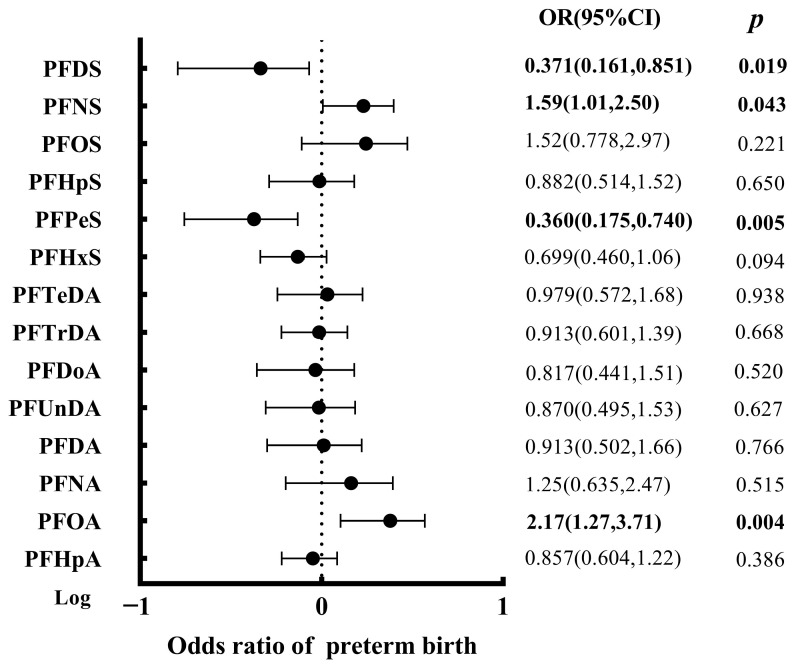
Odds ratios (ORs) [95% confidence interval (CI)] for preterm birth by serum concentrations of PFASs in logistic regression analyses. Notes: preterm birth risk was estimated for continuous log-transformed PFASs concentrations in serum; The model was adjusted for maternal age, prenatal BMI, education, occupation, smoking, alcohol drinking, ethnicity, delivery mode and parity.

Binary logistic regressions were performed to present the associations between each PFASs compound exposure and preterm birth. As Figure 4 demonstrated, the odds of preterm birth was 2.17 fold (95% CI: 1.27, 3.71, *p* = 0.004) and 1.59 fold (95% CI: 1.01, 2.50, *p* = 0.043) greater per log-ng/mL of PFOA and PFNS concentration in maternal serum. This was in accordance with numerous studies, for example, elevated odds of preterm birth were found in association with higher maternal PFOA, PFBA, and PFNA from a family-based birth cohort study in coastal China [33]. Similar results have been reported in Guangzhou with a significant 2.03-fold (95% CI: 1.24, 3.32) higher odds of preterm birth per log-ng/mL PFOS in maternal serum [27]. A nearly 2-fold increase in preterm risks was observed for the higher quartiles of PFOA and PFOS exposure birth from a Danish National Birth Cohort of 3535 mother-infant pairs, higher PFNA, PFHpS, and PFDA also led to the elevated risks of preterm birth [70]. Nevertheless, some of the literature showed contradictory evidence. No significant associations were observed between PFASs and overall spontaneous or indicated preterm birth in 2849 maternal-neonatal pairs in Shanghai, China [34]. Two findings suggested no elevated preterm risk was induced by low PFASs maternal exposure [71,72]. A threshold effect of PFASs at less than 2 ng/mL was less likely to increase preterm risk [72].

Besides, in this study, the associations were reversed for PFPeS (OR = 0.360, 95% CI: 0.175, 0.740, *p* = 0.005) and PFDS (OR = 0.371, 95% CI: 0.161, 0.851, *p* = 0.019), which inhibited the preterm development. The inverse associations were unexpected and we did not find directly comparable results from other studies. Moreover, the joint exposure model indicated an overall inverse dose-response relationship that increasing all PFASs in the mixture by one quartile was similarly associated with a modest reduction in the preterm incidence (OR: 0.356, 95% CI: 0.149, 0.845, *p* = 0.019) (Figure S2d). PFOS, PFOA, and PFNA tended to increase the odds of preterm birth, while others, especially the PFDS and PFPeS, exerted a protective influence in the total PFAS mixture estimate on preterm birth (Figure 4). To date, only two studies considered the combined effect that PFASs were significantly positively associated with the risk of preterm birth, which was consistent within the single exposure analysis [33,48]. It should be noted that comparing our results with these previous studies was challenging due to the incomparable exposure levels, study population, and diversity in the sample matrix. Especially when we collected the maternal serum before delivery as the biomonitoring target. Given concerns about effective dose and reverse causality on the part of this manuscript, whether these PFASs congeners had higher placental transfer efficiency than PFOS and PFOA, which result in the underestimated association with maternal serum concentrations should be further clarified.

## 4. Conclusions

PFASs were widely distributed in the maternal serum, with PFOA, PFOS, and PFHpA the most abundant PFASs. Maternal age, BMI, parity and delivery mode were considered as influencing factors of the PFASs burden. Multivariate linear regression suggested that prenatal exposure to PFOA, PFOS, and PFNS significantly reduced neonatal birth weight. PFHpA and PFHpS exposure was associated with increased Apgar-1 scores. PFOA and PFNS were identified as risk factors to preterm birth.

There are some limitations in the present study including the mixed conclusions with the deficiency of both maternal and neonatal evaluation biomarkers. We brought new insights regarding the occurrence of PFASs in humans and Apgar scores. However, we cannot draw the conclusion that PFASs improve the neonatal development on the Apgar since PFHpA and PFHpS were not major components in the total PFASs exposure. Moreover, the plancenta transfer for PFASs from mothers to fetus is not investigated in this study. In this respect, overarching investigations of the active transport mechanism, prenatal exposure, and reproductive risks of PFASs with a larger sample size are warranted.

## Figures and Tables

**Table 1:** Effect of different demographic characteristics on the distribution of PFASs (ng/mL) in maternal serum (Mean ± SD).

	PFHpA	PFOA	PFNA	PFDA	PFUnDA	PFDoA	PFTrDA
**Maternal age**							
<30	0.045 ± 0.756	**0.957 ± 0.526**	**0.140 ± 0.374**	**0.163 ± 0.399**	**0.091 ± 0.419**	**0.711 ± 0.410**	0.648 ± 0.578
30–35	0.142 ± 0.763	**0.963 ± 0.561**	**0.212 ± 0.366**	**0.192 ± 0.374**	**0.174 ± 0.446**	**0.675 ± 0.411**	0.644 ± 0.642
>35	0.259 ± 0.757	**1.22 ± 0.415**	**0.299 ± 0.364**	**0.340 ± 0.455**	**0.368 ± 0.420**	**0.578 ± 0.372**	0.634 ± 0.514
*p*	0.123	0.000 **	0.003 *	0.003 *	0.000 **	0.043 *	0.986
Prenatal BMI							
<25	0.068 ± 0.726	0.938 ± 0.569	0.217 ± 0.335	0.237 ± 0.354	0.196 ± 0.414	0.650 ± 0.393	0.607 ± 0.555
25–30	0.125 ± 0.773	1.023 ± 0.510	0.190 ± 0.393	0.179 ± 0.406	0.171 ± 0.455	0.687 ± 0.405	0.667 ± 0.629
>30	0.279 ± 0.765	1.098 ± 0.508	0.191 ± 0.388	0.220 ± 0.482	0.136 ± 0.452	0.678 ± 0.446	0.655 ± 0.580
*p*	0.181	0.071	0.758	0.335	0.614	0.663	0.760
Mode of delivery							
Spontaneous labor	0.057 ± 0.748	**0.850 ± 0.543**	0.171 ± 0.368	**0.136 ± 0.360**	**0.109 ± 0.415**	**0.712 ± 0.412**	0.656 ± 0.594
cesarean birth	0.176 ± 0.769	**1.14 ± 0.487**	0.221 ± 0.375	**0.264 ± 0.428**	**0.229 ± 0.453**	**0.640 ± 0.399**	0.633 ± 0.599
*p*	0.110	0.000 **	0.136	0.000 **	0.002 *	0.050 *	0.679
Parity							
1	0.101 ± 0.762	**1.01 ± 0.505**	**0.121 ± 0.368**	**0.134 ± 0.393**	**0.088 ± 0.432**	**0.726 ± 0.396**	**0.719 ± 0.619**
2	0.167 ± 0.769	**0.953 ± 0.584**	**0.211 ± 0.401**	**0.197 ± 0.390**	**0.176 ± 0.450**	**0.658 ± 0.382**	**0.626 ± 0.600**
≥3	0.124 ± 0.758	**1.04 ± 0.517**	**0.295 ± 0.325**	**0.316 ± 0.406**	**0.296 ± 0.415**	**0.610 ± 0.437**	**0.550 ± 0.544**
*p*	0.772	0.002 *	0.000 **	0.000 **	0.000 **	0.028 *	0.037 *
	PFTeDA	PFOS	PFNS	PFHxS	PFPeS	PFHpS	PFDS
Maternal age							
<30	1.21 ± 0.510	**0.563 ± 0.336**	1.18 ± 0.569	0.485 ± 0.722	**1.23 ± 0.358**	**1.19 ± 0.478**	1.28 ± 0.348
30–35	1.13 ± 0.527	**0.671 ± 0.387**	1.19 ± 0.542	0.503 ± 0.749	**1.19 ± 0.453**	**1.08 ± 0.568**	1.25 ± 0.369
>35	1.07 ± 0.537	**0.803 ± 0.366**	1.19 ± 0.416	0.696 ± 0.736	**1.32 ± 0.325**	**0.95 ± 0.453**	1.34 ± 0.321
*p*	0.215	0.000 **	0.972	0.129	0.049 *	0.006 **	0.201
Prenatal BMI							
<25	1.14 ± 0.530	0.660 ± 0.372	1.18 ± 0.530	0.411 ± 0.728	1.24 ± 0.351	1.19 ± 0.519	1.28 ± 0.320
25–30	1.15 ± 0.499	0.643 ± 0.377	1.19 ± 0.543	0.581 ± 0.730	1.19 ± 0.437	1.02 ± 0.528	1.26 ± 0.392
>30	1.14 ± 0.596	0.656 ± 0.370	1.19 ± 0.521	0.579 ± 0.745	1.29 ± 0.353	1.15 ± 0.468	1.32 ± 0.288
*p*	0.221	0.767	0.985	0.546	0.067	0.133	0.501
Mode of delivery							
Spontaneous labor	**1.22 ± 0.487**	**0.586 ± 0.360**	1.23 ± 0.508	**0.365 ± 0.725**	**1.17 ± 0.413**	1.16 ± 0.479	1.25 ± 0.336
cesarean birth	**1.10 ± 0.541**	**0.705 ± 0.374**	1.15 ± 0.550	**0.675 ± 0.721**	**1.27 ± 0.380**	1.05 ± 0.548	1.30 ± 0.368
*p*	0.044 *	0.000 **	0.099	0.000 **	0.004 *	0.057	0.101
Parity							
1	1.19 ± 0.525	**0.612 ± 0.365**	**1.12 ± 0.580**	**0.547 ± 0.737**	1.21 ± 0.379	**1.13 ± 0.522**	1.27 ± 0.346
2	1.14 ± 0.520	**0.644 ± 0.376**	**1.19 ± 0.563**	**0.347 ± 0.714**	1.20 ± 0.393	**1.18 ± 0.450**	1.31 ± 0.340
≥3	1.10 ± 0.525	**0.713 ± 0.375**	**1.27 ± 0.407**	**0.678 ± 0.733**	1.26 ± 0.434	**0.968 ± 0.562**	1.25 ± 0.377
*p*	0.447	0.041 *	0.046 *	0.003 **	0.408	0.008 **	0.371

Note: Nonparametric Mann-Whitney U testing and Kruskal-Wallis analysis were conducted to compare the concentrations of PFASs between different groups of sociodemographic predictors, * *p* < 0.05; ** *p* < 0.01.

**Table 2:** Multivariable linear regression analyses of serum PFASs in relation to birth weight and Apgar scores.

	Birth Weight		Apgar-1		Apgar-5	
Compounds	β (95% CI)	*p*	β (95% CI)	*p*	β (95% CI)	*p*
PFHpA						
Univariate ^a^	0.072 (−0.025, 0.169)	0.145	**0.079 (0.015, 0.142)**	0.016 *	0.004 (−0.013, 0.022)	0.629
Fully adjusted ^b^	0.091 (−0.002, 0.183)	0.056	**0.065 (0.002, 0.129)**	0.044 *	0.002 (−0.016, 0.020)	0.841
PFOA						
Univariate ^a^	**−0.153 (−0.274, −0.031)**	0.014 *	0.049 (−0.128, 0.029)	0.215	−0.001 (−0021, 0.020)	0.960
Fully adjusted ^b^	−0.110 (−0.232, 0.012)	0.077	−0.006 (−0.086, 0.075)	0.887	0.001 (−0.021, 0.024)	0.902
PFNA						
Univariate ^a^	−0.076 (−0.252, 0.100)	0.395	−0.007 (−0.119, 0.105)	0.907	−0.007 (−0.037, 0.023)	0.653
Fully adjusted ^b^	−0.021 (−0.191, 0.149)	0.807	−0.032 (−0.144, 0.081)	0.582	−0.012 (−0.043, 0.019)	0.463
PFDA						
Univariate ^a^	−0.051 (−0.214, 0.113)	0.544	0.005 (−0.099, 0.110)	0.918	0.007 (−0.021, 0.035)	0.631
Fully adjusted ^b^	0.034 (−0.125, 0.194)	0.674	0.010 (−0.096, 0.116)	0.855	0.004 (−0.025, 0.033)	0.772
PFUnDA						
Univariate ^a^	−0.084 (−0.232, 0.064)	0.266	0.034 (−0.061, 0.128)	0.483	0.010 (−0.015, 0.035)	0.425
Fully adjusted ^b^	−0.017 (−0.163, 0.128)	0.814	0.028 (−0.068, 0.124)	0.571	0.008 (−0.018, 0.035)	0.535
PFDoA						
Univariate ^a^	0.004 (−0.161, 0.168)	0.964	0.039 (−0.063, 0.141)	0.455	0.028 (0.000, 0.056)	0.054
Fully adjusted ^b^	0.068 (−0.089, 0.225)	0.396	0.027 (−0.074, 0.129)	0.597	0.027 (−0.002, 0.055)	0.068
PFTrDA						
Univariate ^a^	0.009 (−0.108, 0.125)	0.885	0.001 (−0.074, 0.076)	0.976	−0.021 (−0.032, 0.008)	0.249
Fully adjusted ^b^	0.023 (−0.088, 0.134)	0.684	−0.017 (−0.091, 0.057)	0.654	−0.014 (−0.034, 0.007)	0.182
PFTeDA						
Univariate ^a^	−0.088 (−0.260, 0.084)	0.314	−0.054 (−0.167, 0.059)	0.347	−0.004 (−0.026, 0.017)	0.677
Fully adjusted ^b^	−0.069 (−0.240, 0.101)	0.425	−0.049 (−0.160, 0.063)	0.389	−0.004 (−0.026, 0.017)	0.702
PFHxS						
Univariate ^a^	**0.123 (0.024, 0.223)**	0.015 *	0.057 (−0.008, 0.121)	0.087	0.008 (−0.006, 0.022)	0.258
Fully adjusted ^b^	**0.108 (0.012, 0.204)**	0.028 *	0.040 (−0.024, 0.105)	0.221	−0.006 (−0.008, 0.021)	0.378
PFPeS						
Univariate ^a^	**0.226 (0.053, 0.398)**	0.010 *	**0.139 (0.027, 0.251)**	0.015 *	0.024 (−0.008, 0.056)	0.137
Fully adjusted ^b^	**0.171 (0.008, 0.333)**	0.039 *	**0.117 (0.006, 0.228)**	0.039 *	0.023 (−0.009, 0.056)	0.153
PFHpS						
Univariate ^a^	−0.018 (0.174, 0.138)	0.824	0.069 (−0.036, 0.174)	0.194	0.009 (−0.020, 0.039)	0.528
Fully adjusted ^b^	−0.014 (−0.162, 0.133)	0.849	0.080 (−0.023, 0.184)	0.129	0.011 (−0.019, 0.041)	0.460
PFOS						
Univariate ^a^	**−0.217 (−0.385, −0.049)**	0.012 *	0.008 (−0.104, 0.120)	0.883	0.006 (−0.024, 0.036)	0.678
Fully adjusted ^b^	0.037 (−0.071, 0.145)	0.498	0.073 (−0.043, 0.189)	0.217	0.018 (−0.014, 0.050)	0.272
PFNS						
Univariate ^a^	**−0.137 (−0.270, −0.003)**	0.044 *	−0.024 (−0.107, 0.059)	0.569	−0.011 (−0.028, 0.006)	0.192
Fully adjusted ^b^	0.045 (−0.037, 0.126)	0.284	0.018 (−0.066, 0.102)	0.673	−0.005 (−0.023, 0.012)	0.549
PFDS						
Univariate ^a^	−0.104 (−0.234, 0.026)	0.117	−0.007 (−0.089, 0.075)	0.863	−0.008 (−0.025, 0.008)	0.326
Fully adjusted ^b^	0.171 (−0.018, 0.361)	0.076	0.014 (−0.121, 0.149)	0.836	−0.001 (−0.040, 0.037)	0.951

^a^ The estimate was described as β (95% CI) derived from one quantile increase in overall PFASs mixture. ^b^ The estimates were described as OR (95% CI) derived from one quantile increase in overall PFASs mixture. * *p* < 0.05 was regarded as statistically significant.

**Table 3:** Estimates and 95% CIs for quantile-based g-computation of PFASs on birth outcomes.

PFAS Mixture	Estimates	95% CI	*p* Value
birth weight ^a^	0.096	−0.170, 0.363	0.479
Apgar-1 ^a^	0.324	0.068, 0.579	0.013 *
Apgar-5 ^a^	0.128	−0.083, 0.399	0.234
preterm birth ^b^	0.356	0.149, 0.845	0.019*

Note: The models were adjusted for maternal age, prenatal BMI, education, occupation, smoking, alcohol drinking, ethnicity, delivery mode and parity. ^a^ The estimate was described as β (95% CI) derived from one quantile increase in overall PFASs mixture. ^b^ The estimate was described as OR (95% CI) derived from one quantile increase in overall PFASs mixture. * *p* < 0.05 was regarded as statistically significant.

## Data Availability

Data available on request due to restrictions eg privacy or ethical.

## Supplementary Materials

The following supporting information can be downloaded at: https://www.mdpi.com/article/10.3390/toxics10110650/s1. Figure S1: Directed acyclic graph for selection of covariates; Figure S2: Effect of mixed exposure of PFASs on birth outcomes; Table S1: General Characteristics of the Study Population; Table S2: Internal Surrogate Standard Spiked, Limits of detection (LOD) and Limit of quantification (LOQ) for Target Analytes in Serum Samples; Table S3: MS Parameters of Target Analytes;Table S4: Levels of PFASs (ng/mL) in Maternal Serum; Table S5: Correlation Analysis of PFASs Compounds in Serum. Ref. [73] is cited in the supplementary materials.
