# Supplementary material for: Perfluoroalkyl Mixture Exposure in Relation to Fetal Growth: Potential Roles of Maternal Characteristics and Associations with Birth Outcomes
Source: Toxics. 2022 Oct 28;10(11):650. doi: 10.3390/toxics10110650 (PMC9695392; doi:10.3390/toxics10110650)
Supplement: Supplementary file 1 [file toxics-10-00650-s001.zip › toxics-1972870-supplementary.pdf]

Supporting Information

Perfluoroalkyl Mixture Exposure in Relation to Fetal Growth: Potential Roles of Maternal Characteristics and Associations with Birth Outcomes

Chensi Shen [1,2], Jiaxin Ding [1], Chenye Xu [1,2,*], Long Zhang [3], Shuren Liu [4] and Yonghong Tian [3,*]

[1] College of Environmental Science and Engineering, Donghua University, Shanghai 201620, China
[2] Shanghai Institute of Pollution Control and Ecological Security, Shanghai 200092, China
[3] Women's Hospital, Zhejiang University School of Medicine, Hangzhou 310000, China
[4] Interdisciplinary Research Academy (IRA), Zhejiang Shuren University, Hangzhou 310015, China
* Correspondence: xcy0714@dhu.edu.cn (C.X.); tianyh@zju.edu.cn (Y.T.)

1. Materials and Methods

1.1. Standards and Reagents

Native standards of perfluoroalkyl sulfonic acids (PFSAs) (i.e., PFOS, PFHxS, PFNS, PFDS, PFPeS, PFHpS), perfluoroalkyl carboxylic acids (PFCAs) (i.e., PFOA, PFPeA, PFHpA, PFNA, PFDA, PFUndA, PFDoA, PFTeDA), were all obtained from Wellington laboratories (Guelph, Canada). The corresponding isotopically labeled internal standards including $^{13}C_8$-PFOS, $^{13}C_8$-PFOA, $^{13}C_3$-PFHxS, $^{13}C_7$-PFUndA, $^{13}C_2$-PFDoA, $^{13}C_4$-PFHpA, $^{13}C_9$-PFNA, $^{13}C_5$-PFPeA, $^{13}C_6$-PFDA, $^{13}C_2$-PFDoA, $^{13}C_2$-PFTeDA, and injection standards of $^{13}C_4$-PFOA and $^{13}C_4$-PFOS. The ammonium hydroxide, HPLC grade methanol, and formic acid were purchased from J&k Chemicals (Shanghai, China). Deionized water was prepared by a Milli-Q system and all prepared solutions were stored at 4 ° C.

1.2. Sample Extraction and Instrument Analysis

Specifically, 1 mL serum sample was spiked with 2 ng isotopically labeled internal standard, mixed with 3 mL 2% formic acid in the water. Next, all samples were vortex-mixed for 60 seconds at 400 rmp and sonicated extracted for 20 min. The cartridges (Oasis-WAX cartridges Waters, Milford, MA; 150 mg/6 cc) were activated by washing 4 mL of 0.1% ammonium hydroxide in methanol followed by 4 mL of methanol and 4 mL Milli-Q water. After the precondition, prepared samples were subsequently passed through the cartridges slowly at a flow rate of 1 drops/s (Woudneh et al. 2019). Then, 4 mL of 2% formic acid in the water, 4 mL of 40% methanol in the water (v/v) were utilized to wash the cartridges subsequently at a rate of 1-2 drops/s. The cartridges were dried under vacuum for 30 min followed by eluting chemicals with 3 mL of 1% ammonium hydroxide in methanol and 2 mL methanol at a rate of 0.5 drop/s. The eluates were then collected in 6 mL polypropylene centrifuge tube, evaporated to dryness under a stream of high-purity nitrogen and rediluted in 250 μL methanol (Xu et al. 2019). The extracts mentioned above were vortex-mixedat 2800 rpm followed by being transferred through 0.22 μm microporous membrane with needle syringe to polypropylene vials prior to analysis (Chen et al. 2017).

Compounds were separated using an Acquity UPLC BEH C18 column (2.1 mm × 50 mm, 1.7 μm, Waters, Dublin, Ireland). A 10 μL sample extract was injected into the system. Target compounds were analyzed by performing on a UPLC-tandem electrospray equipped with Xevo TQ-Striple quadrupole mass spectrometry system (Waters ACQUITY UPLC I-Class, Milford, MA)(Kashino et al. 2020). The mobile

phase consisted of Milli-Q water (A) and methanol (B) containing 5 mM ammonium formate in to adjust PH to 3. Analyte chromatographic separation was performed at a flow rate of 0.2 mL/min. The source-dependent parameters in electrospray ionization (ESI) negative mode were set as follows: desolvation temperature was 400 °C; capillary voltage was 3 kV; desolvation gas flow was 1000 L/h; cone gas flow was 150 L/h; collision gas flow was 0.15 mL/min (Gu et al. 2021). Gradient elution was set initially controlled at 50 % B; switched to linear increase to 100% B last for the first 5min; from 5 to 10 min, 100% B; from 10 to 10.5 min, return to to 50% B during 10.5 to 15 min. MS Parameters of Target Analytes are available in **Table S2**.

References:

Chen, F., Yin, S., Kelly, B. C., Liu, W., Isomer-Specific Transplacental Transfer of Perfluoroalkyl Acids: Results from a Survey of Paired Maternal, Cord Sera, and Placentas. *Environ. Sci. Technol.* 2017, ***51*** (10), 5756-5763. http://10.1021/acs.est.7b00268.

Gu, C. J., Xu, C. Y., Zhou, Q., Shen, C. S., Ma, C. Y., Liu, S. R., Yin, S. S., Li, F., Congener- and isomer-specific Perfluorinated compounds in textile wastewater from Southeast China. *J. Clean. Prod.* 2021, ***320***. http://10.1016/j.jclepro.2021.128897.

Kashino, I., Sasaki, S., Okada, E., Matsuura, H., Goudarzi, H., Miyashita, C., Okada, E., Ito, Y. M., Araki, A., Kishi, R., Prenatal exposure to 11 perfluoroalkyl substances and fetal growth: A large-scale, prospective birth cohort study. *Environ. Int.* 2020, ***136***, 105355. http://10.1016/j.envint.2019.105355.

Woudneh, M. B., Chandramouli, B., Hamilton, C., Grace, R., Effect of Sample Storage on the Quantitative Determination of 29 PFAS: Observation of Analyte Interconversions during Storage. *Environ. Sci. Technol.* 2019, ***53*** (21), 12576-12585. http://10.1021/acs.est.9b03859.

Xu, C. Y., Yin, S. S., Liu, Y. X., Chen, F. F., Zhong, Z. H., Li, F., Liu, K., Liu, W. P., Prenatal exposure to chlorinated polyfluoroalkyl ether sulfonic acids and perfluoroalkyl acids: Potential role of maternal determinants and associations with birth outcomes. *J. Hazard. Mater.* 2019, ***380***. http://10.1016/j.jhazmat.2019.120867.

Table S1. General Characteristics of the Study Population (N=506)

**Characteristic index**	**Mean ± SD**	**25th**	**50th**	**75th**	**%**
**Age (years)**	31.3 ± 4.28	28	31	34	
**Prenatal BMI (kg/m^2)**	26.7 ± 3.16	24.4	26.6	28.8	
**Education**					
**below high school**					15.8
**college degree**					71.1
**postgraduate**					13.0
**Occupation**					
**employee**					91.3

**self-employment**					3.9
**unemployment**					4.7
**Smoking**					
**yes**					0
**no**					100
**Alcohol drinking**					
**yes**					0
**no**					100
**Ethnicity**					
**Han**					99.4
**Others**					0.593
**Gestational age (day)**	265 ± 28.3	262	268	274	
**Parity**					
**1**					41.3
**2**					29.2
**3+**					29.4
**Mode of delivery**					
**Spontaneous labor**					51.2
**Cesarean birth**					48.8
**Birth gender**					
**Girls**					47.1
**Boys**					52.9

**Birth weight (kg)**	3.11 ± 0.75	2.95	3.21	3.51	
**APGAR-1**	9.88 ± 0.47	10	10	10	
**APGAR-5**	9.99 ± 0.13	10	10	10	

Note: Data are presented as the "means (±SD)" for continuous variables and "%" for categorical variables

Table S2. Internal Surrogate Standard Spiked, Limits of detection (LOD) and Limit of quantification (LOQ) for Target Analytes in Serum Samples

Compound	Internal standard	Recovery(%) ± SD	LOD (ng/mL)	LOQ (ng/mL)
PFOS	$^{13}C_4$-PFOS	103 ± 5.50	0.005	0.018
PFHxS	$^{13}C_3$-PFHxS	89.7 ± 9.10	0.004	0.013
PFHpS	$^{13}C_4$-PFOS	81.3 ± 5.31	0.004	0.013
PFNS	$^{13}C_4$-PFOS	87.4 ± 6.21	0.008	0.027
PFPeS	$^{13}C_4$-PFOS	86.3 ± 10.3	0.008	0.013
PFDS	$^{13}C_4$-PFOS	80.3 ± 5.81	0.004	0.013
PFDoA	$^{13}C_2$-PFDoA	92.0 ± 5.26	0.009	0.030
PFUnDA	$^{13}C_7$-PFUnDA	95.8 ± 10.8	0.008	0.027
PFDA	$^{13}C_6$-PFDA	86.7 ± 10.0	0.008	0.027
PFNA	$^{13}C_9$-PFNA	99.1 ± 8.95	0.004	0.013
PFOA	$^{13}C_4$-PFOA	97.5 ± 11.3	0.008	0.016
PFHpA	$^{13}C_4$-PFHpA	93.4 ± 12.0	0.004	0.013
PFTrDA	$^{13}C_4$-PFOA	92.8 ± 8.82	0.005	0.013
PFTeDA	$^{13}C_2$-PFTeDA	75.5 ± 7.81	0.004	0.027

Table S3. MS Parameters of Target Analytes

Compound	Precursor ion(m/z)	Production ion(m/z)	Cone voltage (V)	Collision Energy (V)
**Native standards**				
PFHxS	399	80	62	38
PFPeS	349	80	4	30
PFHpS	449	80	4	40
PFOS	499	80	84	44
PFNS	549	80	100	42
PFDS	599	80, 99	76	50, 44
PFHpA	363	319, 169	20	10, 18
PFOA	413	369	6	10
PFNA	463	419, 169	20	10, 18
PFDA	513	469, 219	20	10, 20
PFUnDA	563	519, 269	20	12, 18
PFDoA	613	569, 169	25	10, 28
PFTrDA	663	619, 169	22	12, 28
PFTeDA	713	669	30	15
**Internal standards**				
$^{13}C_8$-PFOS	507	99	40	40
$^{13}C_4$-PFHpA	367	322	31	14
$^{13}C_8$-PFOA	421	376	10	10

$^{13}C_9$-PFNA	472	427	30	13
$^{13}C_6$-PFDA	519	474	41	13
$^{13}C_7$-PFUnDA	570	525	37	17
$^{13}C_2$-PFDoA	615	570	43	18
$^{13}C_2$-PFTeDA	715	670	44	18
$^{13}C_5$-PFHxA	318	273	21	14
**Injection standards**				
$^{13}C_4$-PFOS	503	80	84	44
$^{13}C_4$-PFOA	417	372	10	10

Table S4. Levels of PFASs (ng/mL) in Maternal Serum

Compounds	Detection frequency (%)	Mean± SD	Percentiles				
			5th	25th	50th	75th	95th
**PFHpA**	82.6	3.78±11.5	0.050	0.250	0.625	2.41	18.3
**PFOA**	99.8	18.5±19.6	1.22	3.74	13.6	26.8	52.1
**PFNA**	92.9	2.27±2.77	0.364	0.950	1.66	2.84	5.60
**PFDA**	97.8	2.71±5.33	0.400	0.875	1.48	2.76	7.90
**PFUnDA**	99.0	2.52±3.35	0.275	0.775	1.33	3.16	8.80
**PFDoA**	96.2	0.33±0.43	0.050	0.125	0.200	0.375	1.14
**PFTrDA**	92.7	1.23±10.7	0.025	0.075	0.225	0.500	2.26
**PFTeDA**	61.9	0.36±3.41	0.025	0.025	0.050	0.125	0.990
**PFHxS**	76.9	1.23±2.70	0.025	0.100	0.250	1.08	6.29
**PFOS**	98.0	6.76±9.38	1.10	2.69	4.32	7.20	17.9
**PFDS**	80.2	0.09±0.24	0.025	0.025	0.050	0.093	0.200
**PFPeS**	86.2	0.11±0.43	0.025	0.025	0.050	0.100	0.325
**PFHpS**	73.3	0.60±7.68	0.025	0.025	0.075	0.175	0.560
**PFNS**	87.4	0.26±1.07	0.025	0.025	0.050	0.100	1.03

Table S5. Correlation Analysis of PFASs Compounds in Serum

	PFHpA	PFOA	PFNA	PFDA	PFUnDA	PFDoA	PFTrDA	PFTeDA	PFHxS	PFPeS	PFHpS	PFOS	PFNS	PFDS
PFHpA	1.000													
PFOA	-0.021	1.000												
PFNA	0.079	0.146**	1.000											
PFDA	0.098*	0.226**	0.585**	1.000										
PFUnDA	-0.072	0.314**	0.516**	0.763**	1.000									
PFDoA	0.049	0.133**	0.462**	0.667**	0.629**	1.000								
PFTrDA	0.373**	0.034	0.281**	0.359**	0.238**	0.311**	1.000							
PFTeDA	0.041	0.108	0.041	0.202**	0.200**	0.188**	0.154**	1.000						
PFHxS	0.080	-0.463	0.017	-0.126	-0.103	-0.088	-0.035	-0.033	1.000					
PFPeS	0.148**	-0.271	0.030	-0.071	-0.075	-0.004	0.161**	-0.027	0.242**	1.000				
PFHpS	0.027	0.349**	0.130*	0.181**	0.321**	0.209**	0.284**	0.076	-0.183	0.163**	1.000			

PFOS	-0.072	0.353**	0.488**	0.695**	0.669**	0.549**	0.181**	0.248**	-0.062	0.055	0.337**	1.000		
PFNS	0.116*	0.187**	0.031	0.027	-0.149	0.029	0.150**	-0.046	-0.164	0.098	-0.172	0.189**	1.000	
PFDS	0.121*	-0.095	0.083	0.148**	0.110*	0.157**	0.212**	0.079	0.128*	0.297**	0.298**	0.232**	0.107*	1.000

$^{*}p<0.05$; $^{**}p<0.01$

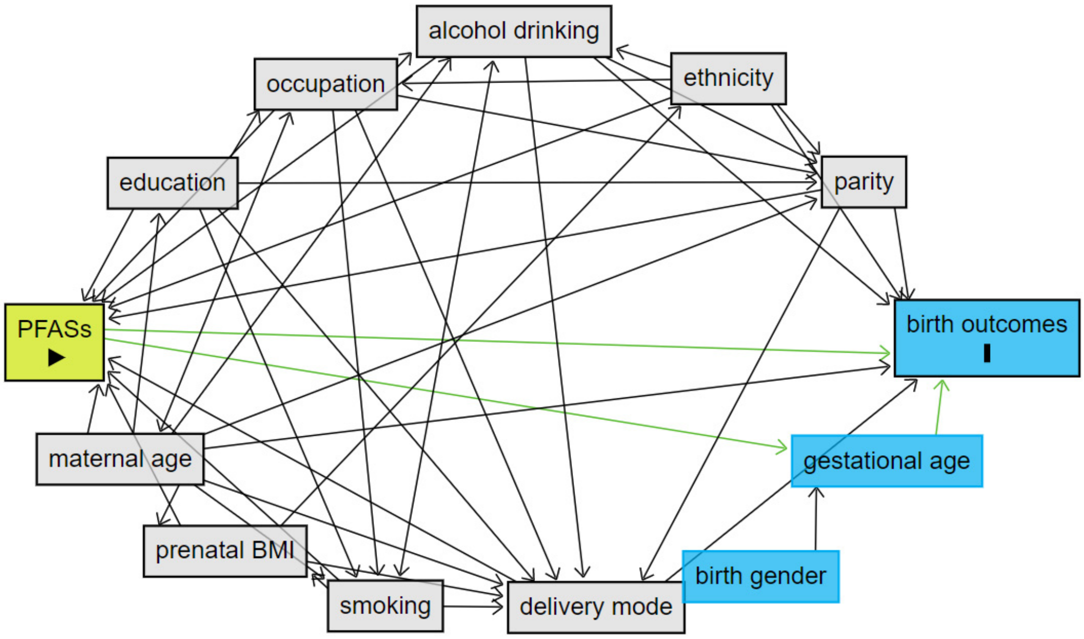

Figure S1. Directed acyclic graph for selection of covariates.

Color legend: 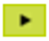 **: PFASs exposure;** 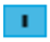 **:outcomes;** 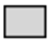 **: covariates included in the multivariate models;** 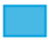 **: ancestors of outcome;**

Green line: presumed causal path.

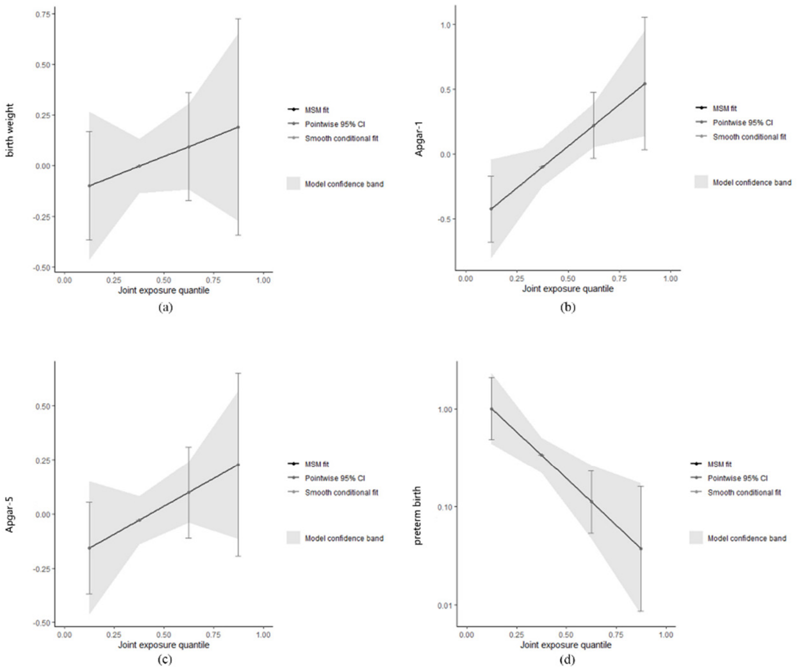

Figure S2. Effect of mixed exposure of PFASs on birth outcomes. (a) birth weight, (b) Apgar-1, (c) Apgar-5, (d) preterm birth. Note: PFAS were log transformed and missing data imputed. The model was adjusted for maternal age, prenatal BMI, education, occupation, smoking, alcohol drinking, ethnicity, delivery mode and parity.
